# A longitudinal assessment of retinal function and structure in the APP/PS1 transgenic mouse model of Alzheimer’s disease

**DOI:** 10.1186/s40035-019-0170-z

**Published:** 2019-10-01

**Authors:** Dana Georgevsky, Stephanie Retsas, Newsha Raoufi, Olga Shimoni, S. Mojtaba Golzan

**Affiliations:** 1 Vision Science group, Graduate School of Health (Orthoptics Discipline), University of Technology Sydney, 15 Broadway, Ultimo, Sydney, NSW 2007 Australia; 2 Institute of Biomedical Materials & Devices (IBMD), Faculty of Science, University of Technology Sydney, 15 Broadway, Ultimo, Sydney, NSW 2007 Australia

**Keywords:** Retinal function, Retinal structure, Retinal imaging, Biomarkers, Alzheimer’s disease, Early assessment

## Abstract

**Background:**

A great body of evidence suggests that there are retinal functional and structural changes that occur in Alzheimer’s disease (AD). However, whether such changes are primary or secondary remains to be elucidated. We studied a range of retinal functional and structural parameters in association with AD- specific pathophysiological markers in the double transgenic APP/PS1 and control mice across age.

**Methods:**

Electroretinogram (ERG) and optical coherence tomography (OCT) was performed in APP/PS1 and wild type (WT) control mice every 3 months from 3 to 12 months of age. For functional assessment, the a- and b-wave of the ERG, amplitude of oscillatory potentials (OP) and the positive scotopic threshold response (pSTR) were quantified at each time point. For structural assessment, the inner and outer retinal thickness was segmented and measured from OCT scans. Episodic memory was evaluated at 6, 9 and 12 months of age using the novel object recognition test. Amyloid beta (Aβ) distribution in the hippocampus and the retina were visualised at 3, 6 and 12 months of age. Inter- and intra- group analysis was performed to study rate of change for each parameter between the two groups.

**Results:**

Inter-group analysis revealed a significant difference in b-wave and OPs of APP/PS1 compared to WT controls starting from 3 months (*p* < 0.001). There was also a significant difference in the amplitude of pSTR between the two groups starting from 6 months (*p* < 0.001). Furthermore, a significant difference in the inner retinal thickness, between the two groups, was observed starting from 9 months (*p* < 0.001).

**Conclusions:**

We observed an age-related decline in retinal functional and structural parameters in both APP/PS1 and WT controls, however, inter-group analysis revealed that inner retinal functional and structural decline is exacerbated in APP/PS1 mice, and that retinal functional changes precede structural changes in this strain. Further studies are required to confirm whether such phenomenon occurs in humans and if studying retinal functional changes can aid-in early assessment of AD.

**Electronic supplementary material:**

The online version of this article (10.1186/s40035-019-0170-z) contains supplementary material, which is available to authorized users.

## Background

Alzheimer’s disease (AD) is a chronic and progressive neurodegenerative disease affecting nearly 44 million people worldwide. It is the most common form of dementia with cognitive impairment as the main clinical manifestation [1]. Currently, diagnosis is primarily based on patient history, neuropsychological testing and in some cases neuroimaging, including positron emission tomography (PET) scans and magnetic resonance imaging (MRI). Neuroimaging methods are expensive, invasive and still not completely conclusive [2]. The challenge with a pre –mortem diagnosis is the current inability to determine whether the pathological hallmarks of AD are firstly detectable. Furthermore, the pathological changes occurring in the brain from early stages through to disease progression cannot be monitored. Extracellular amyloid-beta (Aβ) plaques and intracellular neurofibrillary tangles are the hallmark proteins of AD and currently are only visible on post mortem histopathological analysis [3]. However, patients presenting with complaints of cognitive decline have a substantial amount of damage that has already occurred for a long time. There is evidence in the literature that initial pathophysiological processes commence as early as 20 years prior to clinical symptoms, indicating a large latency period from pre-symptomatic to symptomatic AD [4]. As a result, it has been reported that up to 15% of patients have received an incorrect diagnosis of “probable” AD [5]. Clearly, having a definite diagnosis that is only possible following a post-mortem histological analysis, urgently warrants further investigation into identifying potential early biomarkers of pre-symptomatic AD [6].

A growing body of evidence suggests sensory disturbances as a common complaint in patients with AD [7]. The visual system and more specifically the retina, has received much attention in identifying AD-specific pathogenesis that can aid-in staging the very early phases of the disease [8–11]. Retinal dysfunction in both human studies of AD patients as well as animal models of AD suggests a plausible link between the retina and brain [12–15]. We have previously reported retinal changes in 75 participants with subjective memory complaint and found a significant correlation between thinning of the retinal nerve fibre layer (RNFL), vascular dysfunction and higher neocortical Aβ scores [16]. These results were consistent with other reports which have also shown retinal structural changes, such as RNFL thinning and retinal ganglion cell (RGC) loss in AD patients compared with aged matched controls [17, 18]. A study using 13–16 month old APP/PS1 mice reported an increase in amyloid deposition in the retina, specifically in close proximity to retinal micro vessels, which corresponded with the levels detected in the brain [15]. The same study reported an inner retinal functional deficit in the AD mice compared to control mice.

The retinal abnormalities seen in AD and its’ origin or impact are still unclear. However, it has been suggested that the structural and functional changes observed in the retina, such as RNFL thinning, RGC loss, and compromised inner retinal function, may be due to neurotoxicity effects from the Aβ plaques [19, 20]. With the majority of these studies reporting their findings in participants/animals with established disease pathology, it remains unclear whether retinal manifestations precede cerebral pathology or occur in parallel or following AD onset. There is limited evidence in the literature with regards to the timing of retinal changes observed in AD, with one study showing amyloid plaques in the retina as early as 2.5 months of age compared with 5 months in the brain [8]. The current study aims to explore this further by investigating markers of retinal structure and function in association with behavioural and neuropathological changes of the amyloid precursor protein- Presenilin 1 (APP/PS1) mice from 3 to 12 months of age. More specifically, we studied an array of electroretiongram parameters and retinal thickness (both inner and outer retina) in association with progressive AD-specific neuropathological changes in Aβ levels. Furthermore, we investigated the potential link between these parameters and levels of cognitive impairment, assessed by the novel object recognition test. Ultimately, our aim is to clarify whether retinal changes are observed prior, parallel or post neuropathological changes in the APP/PS1 mouse model of AD.

## Methods

### Animals

APP/PS1 mice were re-derived at the Australian phonemics facility (ANU Canberra). Genotyping was performed, and wild type (WT) littermates were used as controls. The genotype ratio was approximately 50:50 with mixed genders. At 12 weeks of age, the mice were transported to the University of Technology Sydney and housed in a temperature-controlled facility. Animals were on a 12-h light/dark cycle and were given ad lib access to food and water as per the facilities standard operating procedures. All animal experiments were conducted in accordance with the Australian code of practice for the care and use of animals for scientific purposes. Experimental work was approved by UTS Animal Ethics Committee prior to the commencement of experiments (ETH16–0246). APP/PS1 and WT littermates (*n* = 70, mixed sexes roughly 50:50) were followed longitudinally from 3 to 12 months with time-point data captured every 3 months (ie 3, 6, 9, and 12). For all experiments, animals were anaesthetised with an intraperitoneal injection of ketamine (75 mg/kg) and xylazine (10 mg/kg). The effects of xylazine were reversed with a subcutaneous injection of atipamezole (0.75 mg/kg).

### Optical coherence tomography (OCT)

Retinal structural changes were assessed using OCT at each time-point (spectral domain OCT (Wasatch Photonics, USA- field of view: > 40^o^, central wavelength: 800 nm, axial resolution: 3.9 μm, transverse resolution: 4 μm and imaging rate: 20 Hz). Animals were anaesthetised as described previously and a drop of 1% tropicimide (Alcon laboratories, Australia) was applied to each eye to dilate the pupil. Animals were placed on a heating pad during imaging to maintain ambient body temperature. Ophthalmic gel was applied to the surface of the eyes to prevent dryness from anaesthesia as well as maintaining contact between the cornea and the camera lens.

#### Scanning protocol

**Fig. 1:**
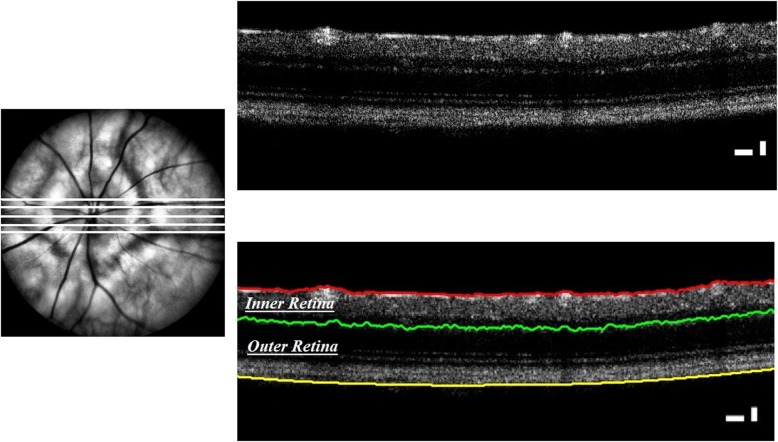
OCT of the mouse optic nerve head. Top Left) five B-scans obtained across the optic nerve head, Top Right) a sample radial OCT scan, Lower Right) results of segmenting the OCT scan into inner and outer retina

The scanning protocol included a 3 × 3 mm perimeter square image of the fundus. The device or the animal were adjusted in such way that the optic nerve head (ONH) was centred on the image. To obtain a raster scan of the ONH, the fundus image was transected across the centre of the ONH to produce a B-scan image of the retinal layers. A further two parallel B-scans on either side of the original B-scan (125 μm apart) was obtained (Fig. 1a).

#### Image segmentation

A modified version of the segmentation algorithm developed by Chiu SJ et al [21] based on graph theory was used to segment the retina into two sections; inner and outer retina. We observed a segmentation error of less than 5% across all the scans in which we corrected manually. The mean thickness of the five B-scans was used to measure inner retinal thickness (between the ganglion cell layer (GCL) and inner nuclear layer (INL) and outer retinal thickness (outer plexiform layer (OPL) and retinal pigment epithelium (RPE)) (Fig. 1).

### Electroretinogram (ERG)

**Fig. 2:**
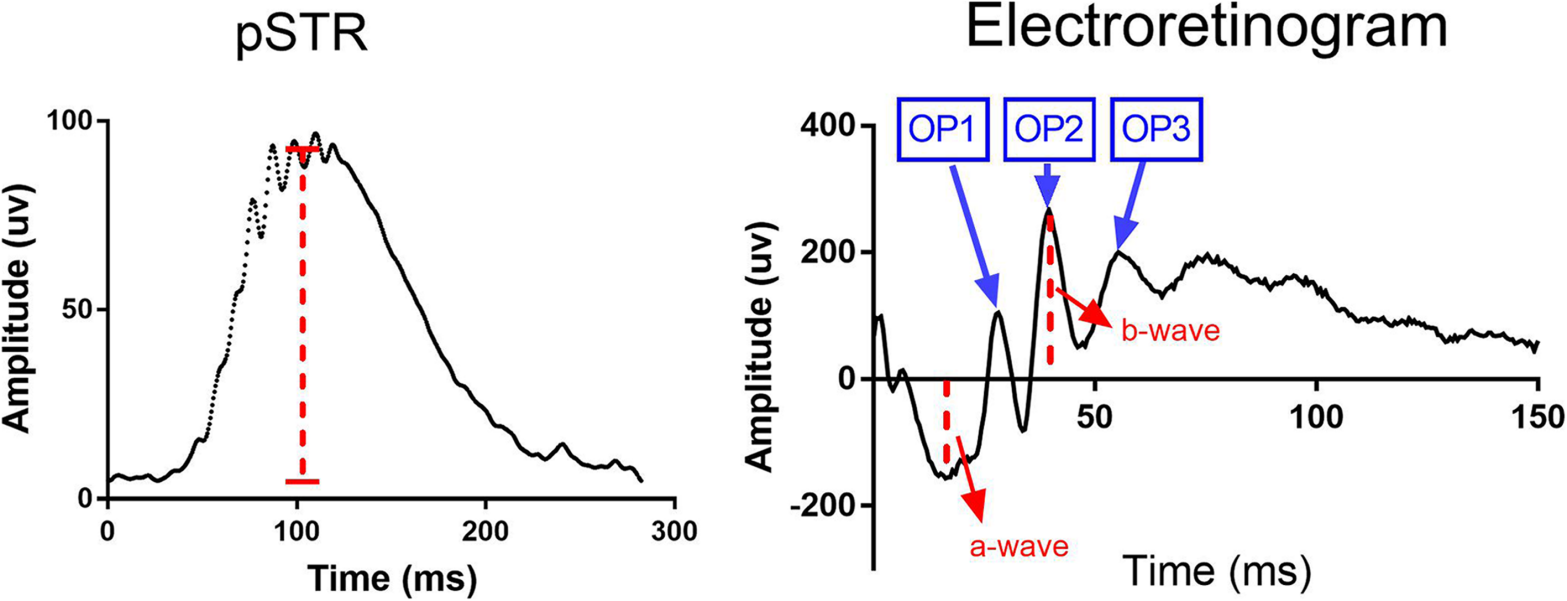
ERG measurements. Right) STR trace and pSTR measurement, Left) ERG trace and a-, b-wave and OP measurements

Animals were dark-adapted overnight prior to the recordings. A dim red light was used to conduct the experiments the following day. Animals were anaesthetised as described previously and placed on a thermostatically controlled heat pad. Both eyes were dilated using a drop of 1% tropicimide following general anaesthesia. The animals were placed on the heat pad of the ERG instrument (Ocuscience, USA) and two silver corneal electrodes were gently placed on the cornea and were kept in position by placing a contact lens on top. Stainless steel reference electrodes were inserted subcutaneously on the forehead of the mouse and at the base of the tail. The Scotopic Threshold Response (STR) was measured first and consisted of the mean of 30 dim flashes (3 cd.s.m-2) with 2-s intervals. This was followed by a single bright flash ERG (30 cd.s.m-2). The positive STR (pSTR) amplitude was measured from the baseline to the maximum peak of the waveform at the flash intensity. The b-wave amplitude was measured from the trough of the a-wave to the peak of the b-wave (Fig. 2).

#### Measurement of oscillatory potentials (OPs)

The OPs were isolated from the intact ERG recordings from each eye. To mitigate effects of a-wave contaminants in early OPs, a digital band pass filter (60-235 Hz) was used (MATLAB). The OP response was determined by the summed amplitude of each OP trace (OP1, OP2, OP3) by a blinded examiner. The amplitude (in microvolts) of each OP was characterised by the difference between the peak and trough. The overall OP response was determined through the summation of each of the OP amplitudes (i.e OP=OP1 + OP2 + OP3).

### Tissue collection

A subgroup of animals were euthanized at 3, 6 and 12 months using an intraperitoneal injection of 50% diluted lethabarb and saline. The eyes were enucleated and placed in 4% paraformaldehyde (PFA) overnight for fixation. The brain was dissected and followed the same regime as the eyes. The fixed tissues were removed from PFA the following morning and washed in phosphate buffered saline (PBS) 3 times × 5 min. The tissues were then placed in 70% ethanol and were processed and embedded in paraffin wax within a week. The paraffin blocks were cut using a microtome at 5 μm thickness. A 1% and 2% Thioflavin S stain was applied on the sections to detect Aβ in the brain’s hippocampus region and retina (within 200 μm of the optic nerve head), respectively. All slides were imaged using an upright fluorescent microscope at 460 nm excitation.

### Novel object recognition

The mice underwent the novel object recognition behavioural assessment at 6, 9 and 12 month time-points. The mice were acclimatized to the testing equipment daily for 1 week prior to the experiment. The animals were placed for 5 min a day in the test box in their holding room to become comfortable with the equipment prior to testing, to limit unwanted behavioural responses from fear/anxiety. The experiments took place in the holding room to avoid stress caused by new/different smells and sounds in unfamiliar rooms. Two identical objects were placed in a black box at opposite ends, and each mouse was allowed 3 min to explore the objects (familiarisation phase). The objects and box were cleaned with 80% ethanol between each animal to alleviate smells that may distract the next animal from the test. One hour later, one of the objects was replaced with a new “novel” object (test phase). Again each mouse was given 3 min to explore the objects. The recordings were then watched by the same person to limit data variability. The *D*^2^ discrimination index was used to present results. The *D*^2^ index is a common measure used to discriminate novel and familiar objects, and is considered as a reliable measure as it corrects for the total exploratory activity of each animal [22]. It is calculated based on the following equation:

$$ {\boldsymbol{D}}^{\mathbf{2}}=\frac{\left(\boldsymbol{novel}-\boldsymbol{familiar}\right)}{\left(\boldsymbol{novel}+\boldsymbol{familiar}\right)} $$

The *D*^2^ index, therefore, corrects for total exploratory behaviour of each animal.

### Statistical analysis

Statistical analysis was performed using Graphpad Prism 7 (San Diego, CA, USA). Data was presented as the mean sum of the total ± standard deviation (SD). Within each group (intra-analysis), a one-way analysis of variance (ANOVA) was applied to assess the overall change. Tukeys post-hoc analysis was used for multiple comparisons within each group. Analysis of covariance using linear regression analysis (ANCOVA) was applied to assess whether the slope of change over time is different between the two groups. An independent students t-test was used to compare the mean difference for each parameter between APP/PS1 and WT mice at each time point. Statistically significant differences were considered at *p*-value < 0.05 and 95% CI.

## Results

### Retinal functional parameters are exacerbated in APP/PS1 mice starting from 3 months

The ERG can be used to assess the functionality of various cells in the retina through an evaluation of the electrical impulse recorded at the cornea in response to a flashlight. The major known parameters of an ERG are the a- and b –wave, scotopic threshold response (STR) and the oscillatory potentials (OP). The a-wave, the initial negative wave recorded following a bright stimulus, is generated as a result of photoreceptor phototransduction. The b-wave, the positive wave following the a-wave, is generated as a result of rod bipolar cells’ depolarization [23] in the dark-adapted retina. The positive STR (pSTR) is known as the most sensitive response of the dark-adapted ERG [24]. pSTR’s originate from the inner retina and are specifically used to study RGC functionality. In addition to STR, the oscillatory potentials (OP) of the ERG is also shown to originate from the inner retina, mainly the inner plexiform layer (IPL) [25]. We assessed and quantified all these parameters (i.e. a-,b- wave, pSTR and total OP) in both of the animal groups across age. The intra-group analysis showed a significant decline in b-wave, pSTR and total OP amplitude and a non-significant increase in the a-wave amplitude of both APP/PS1 and WT controls from 3 to 12 months (one-way ANOVA). A summary of these measurements and their statistical significance are shown in Table 1.

**Table 1:** Age-associated retinal functional changes in APP/PS1 and WT mice*

Time-point (months)	a-wave (μv)	b-wave (μv)	pSTR (μv)	Total OP (μv)
**APP/PS1**				
3	− 157.6 ± 38	217.5 ± 40a,c	123 ± 22a,b,d	173.5 ± 29b,d
6	−136.7 ± 28	174.7 ± 35	77.1 ± 15a,b	119.3 ± 29b
9	− 118.9 ± 38	146.2 ± 43a	66.5 ± 8b	92.9 ± 30d
12	−116 ± 57	107.7 ± 37c	49.8 ± 12d,b	98.6 ± 24d
*p-value (ANOVA)*	*0.1*	*< 0.001*	*< 0.0001*	*< 0.0001*
**WT**				
3	− 144.5 ± 45	174.6 ± 31a	112.9 ± 9b,c	117.1 ± 29b,c
6	−144.3 ± 30	155 ± 21a	99.4 ± 18 a	109.1 ± 23a,b
9	− 135.4 ± 29	137.6 ± 34a	83.4 ± 16b,a	80.7 ± 24b,a
12	− 123.2 ± 33	118 ± 31c	74.8 ± 11c,a	68.5 ± 18c,b
*p-value (ANOVA)*	*0.2*	*< 0.001*	*< 0.001*	*< 0.001*

*Tukeys post-hoc analysis. Shared subscripts represent statistically significant differences: a = *p* < .05, b = *p* < .01, c = *p* < .001, d = *p* < .0001

**Fig. 3:**
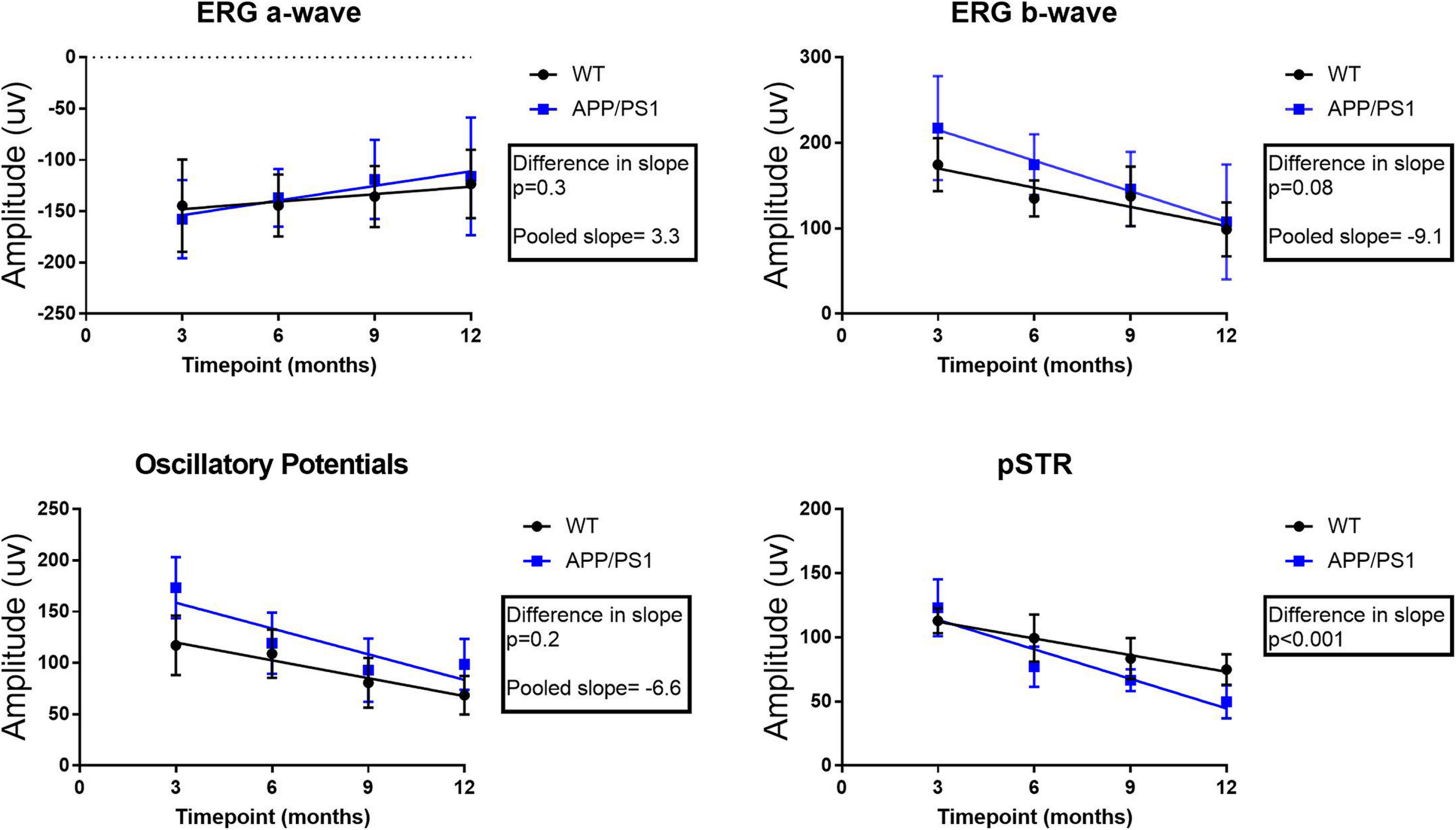
Slope analysis (ANCOVA) for all functional parameters. A significant difference in the slope of decline was only observed in pSTR, suggesting that this parameter’s decline is exacerbated in the APP/PS1 group (*n* = 15 per group, per time-point)

We then assessed whether the slope of change in each parameter over the given time period was significantly different between the APP/PS1 and WT mice. The ANCOVA analysis revealed that for a-, b- wave and OPs, if the overall slopes were identical, there is a 34.7, 8.3 and 24.2% chance, respectively, for randomly choosing data points with slopes this different. This suggests that the differences between the slopes (i.e. rate of change) are not significant (*p = 0.3 [a-wave], p = 0.08 [b-wave], p = 0.2 [OP]*). However, a similar analysis applied to pSTR, revealed that there is a 0.51% chance randomly choosing data points with slopes this different, suggesting the differences between the slopes are very significant (*p* < 0.001) (Fig. 3).

We studied differences in each parameter between the two groups at any given time point. The inter-group analysis revealed a significant difference in b-wave and OPs of APP/PS1 compared to WT controls at 3 months (*p* < 0.001). There was also a significant difference in pSTR between the two groups starting from 6 months (*p* < 0.001). No difference was observed in the a-wave between the two groups at any of the time-points (*p* = 0.1).

### Retinal structural changes are exacerbated in APP/PS1 mice starting from 9 months

We studied changes to the inner (NFL/INL) and outer retinal (OP/RPE) thickness separately to determine whether variations in each structure are associated with the functional changes reported earlier. The intra-group analysis revealed a significant decline in the inner retinal thickness of APP/PS1 and WT control mice from 3 to 12 months. A similar significant change in the outer retinal thickness of APP/PS1 mice was observed, however, the decline in the outer retinal thickness of WT mice was in-significant. A summary of these measurements and their statistical significance are shown in Table 2.

**Table 2:** Age-associated retinal structural changes in APP/PS1 and WT mice*

Time-point (months)	Inner retina	Outer retina
**APP/PS1**		
3	31.2 ± 0.8b,d	54.7 ± 1.4a,b
6	30 ± 1.1b	53 ± 1.9
9	29 ± 1b	52.1 ± 2a
12	28.5 ± 0.7d,b	51 ± 2.3b
*p-value (ANOVA)*	*< 0.0001*	*< 0.01*
**WT**		
3	31.3 ± 0.7a,b	54.1 ± 1
6	30.5 ± 0.9	53.9 ± 1.7
9	30.4 ± 0.7a	53.5 ± 1.5
12	30.1 ± 0.9b	53.1 ± 1
*p-value (ANOVA)*	*< 0.01*	*0.1*

*Tukeys post-hoc analysis. Shared subscripts represent statistically significant differences: a = *p* < .05, b = *p* < .01, c = *p* < .001, d = *p* < .0001

**Fig. 4:**
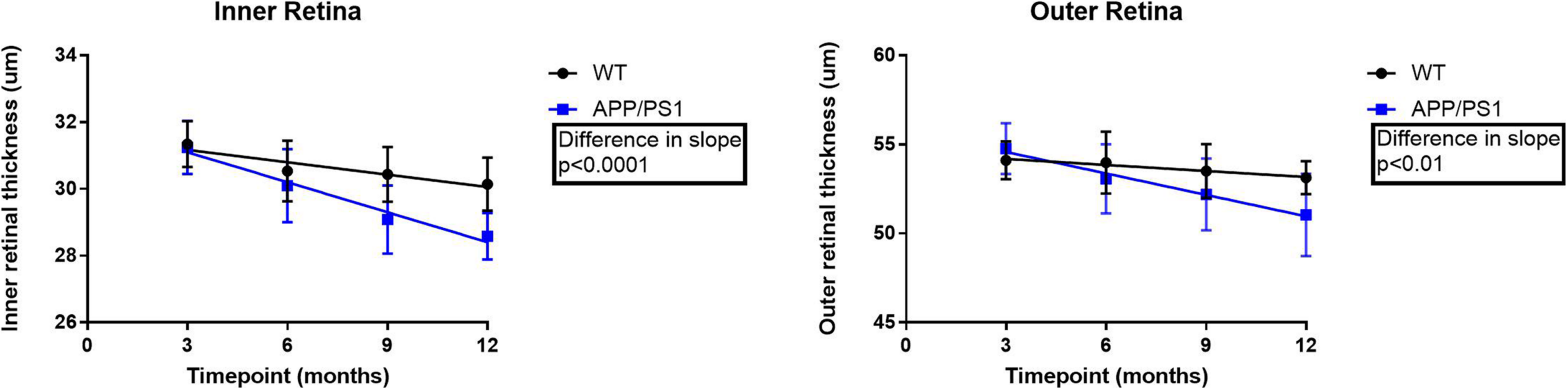
Slope analysis (ANCOVA) for all inner and outer retinal thickness. A significant difference in the slope of decline was observed in both outer and inner retinal thickness, suggesting that structural damage is exacerbated in the APP/PS1 group (*n* = 15 per group, per time-point)

Similar to the retinal function, we assessed whether the slope of change in inner and outer retinal thickness over the given time period was significantly different between the APP/PS1 and WT mice. For inner and outer retinal thickness, results from the ANCOVA analysis revealed that if the overall slopes were identical, there is a 0.09, and 0.5% chance, respectively, for randomly choosing data points with slopes this different. This suggests that the differences between the slopes (i.e. rate of change) are significant (*p < 0.0001 [inner retina], p < 0.01 [outer retina]*) (Fig. 4).

Finally, the inter-group analysis revealed a significant difference in the inner retinal thickness of APP/PS1 compared to WT controls starting at 9 months (*p* < 0.001). There was also a significant difference in the outer retinal thickness between the two groups at 12 months (*p* < 0.01).

### Aβ plaques were observed in the hippocampus and retina of APP/PS1 starting from 3 and 6 months, respectively

**Fig. 5:**
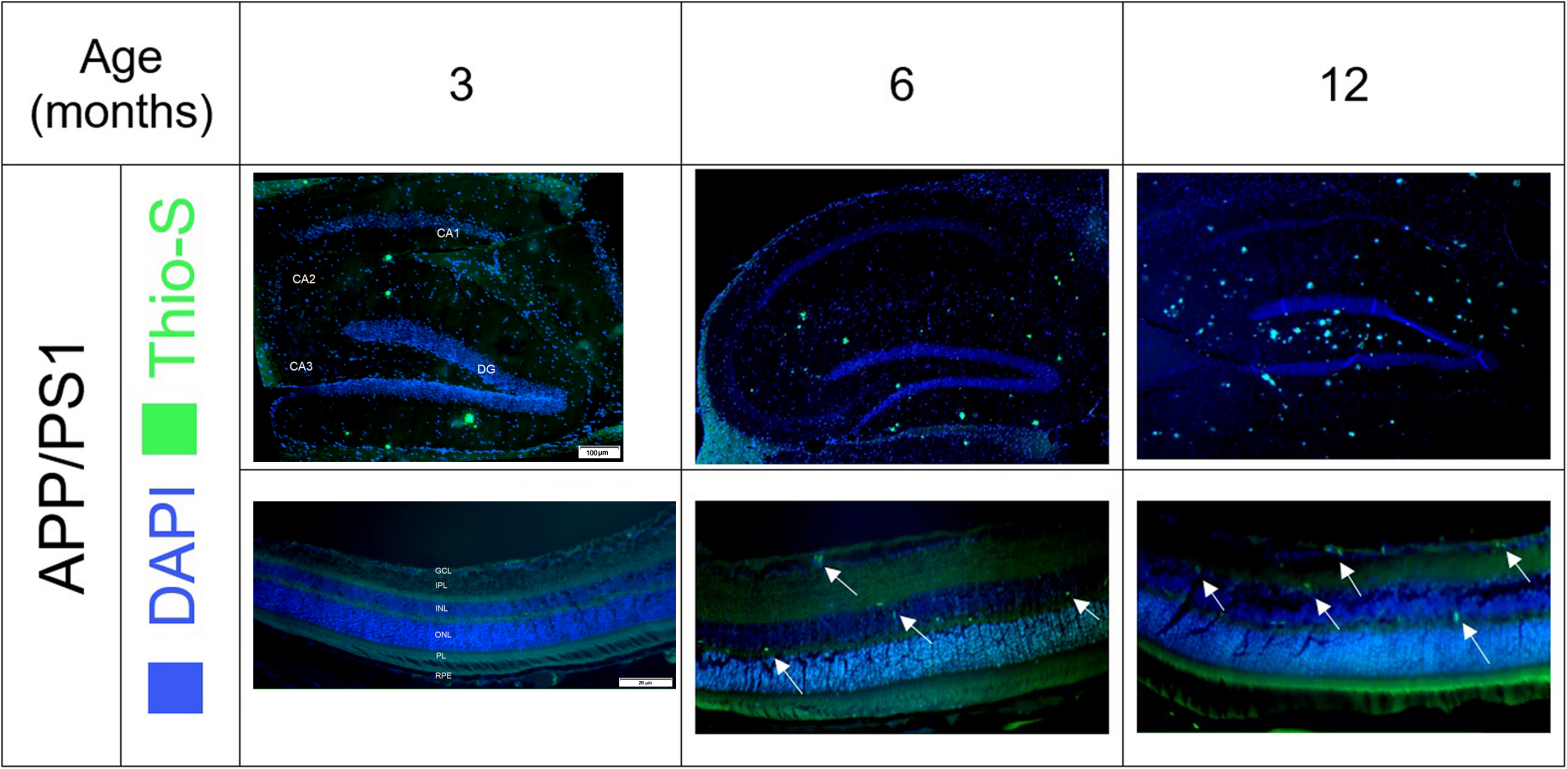
Aβ distribution in the hippocampus and retina of the APP/PS1 mice at 3,6,12 months of age (Dentate Gyrus, CA1-CA3 regions of the hippocampus and retinal layers are shown)- Scale bar for hippocampus and retina is 100 μm and 20 μm, respectively. An increase in Aβ levels in both regions were observed. Retinal Aβ was observed in the inner/outer retinal regions from 6 months and was more pronounced in the GCL by 12 months of age. (Images for WT in Additional file 1: Figure S1)

At 3, 6 and 12 months, five animals from each group was randomly selected, euthanized and sagittal sections of the retina and hippocampus imaged to determine Aβ distribution. Aβ was present in the hippocampus from 3 months and appeared in the retina, mainly in the inner nucleus layer (INL) from 6 months of age. Aβ distribution increased with ageing in the hippocampus and inner retina, mainly ganglion cell layer (GCL), in the APP/PS1 mice (Fig. 5). In contrast, no Aβ was observed in the WT group (Additional file 1: Figure S1).

### No significant change in cognition from 6 to 12 months of age

**Fig. 6:**
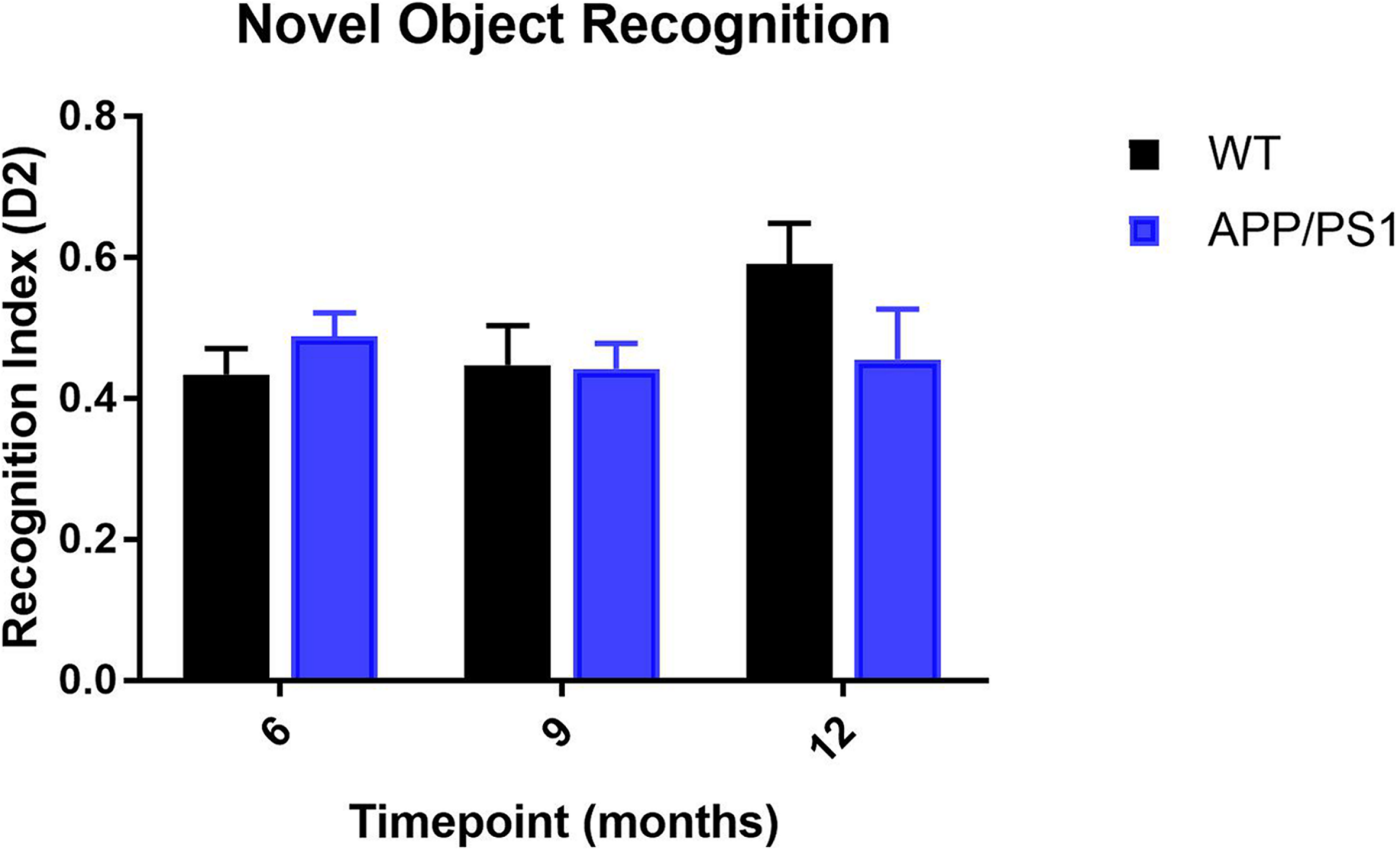
Episodic memory assessment of APP/PS1 and WT mice from 6 to 12 months of age. A non-significant change in the *D*^2^ discrimination index was observed in both group of animals (*n* = 15 per group, per time-point, One-way ANOVA)

We observed a non-significant decline in the D^2^ index of APP/PS1 and a non-significant increase in the D^2^ index of WT mice, from 6 to 12 months (Fig. 6). ANCOVA analysis also revealed a non-significant difference between the sloped of the two groups (Additional file 1: Figure S2).

## Discussion

In this study we investigated an array of retinal functional and structural changes in the APP/PS1 double transgenic mouse model of AD and WT control mice over 12 months. Our results suggest that there is an overall age-dependent decline in retinal functional and structural parameters, however, inner retinal functional and structural damage seems to be accelerated in the APP/PS1 group. More specifically, we observed a significant difference in pSTR amplitude between APP/PS1 and WT controls starting from 6 months with a significant inner retinal thinning following in the APP/PS1 from 9 months. To our knowledge, this is the first study to characterise major functional and structural parameters in the retina of the APP/PS1 mice, longitudinally.

The ERG is used to assess various retinal cellular response to a light stimulus. Consistent with previous reports [26, 27], we found a significant age-dependent decline in ERG parameters in both groups. A significant reduction in pSTR amplitude and ERG latency has also been reported in the symptomatic stage of the same strain previously [15, 28] . We studied ERG parameters in both groups over 12 months and found that the rate of decline was only significant in the pSTR of the APP/PS1 compared to WT controls. This suggests that the disease phenocopy has significantly altered RGC functionality. This is somewhat not surprising, as RGC loss is a known retinal pathology of AD [29]. When ERG parameters were compared between the two groups for any given time-point, we found a significant difference in the outer retina parameters early on in the disease process (i.e. 3 months) with inner retinal function decline to reach significant status only from 6 months. While a reduction in outer retinal ERG amplitude has been reported in AD patients [30], we could not find any other study that has specifically characterised ERG changes in early AD or during mild cognitive impairment. This led us to hypothesise that the significant difference in outer retinal parameters may be attributed to a different starting baseline recording.

There are several studies that have investigated retinal structural changes and more specifically retinal nerve fibre layer (RNFL) thickness in AD [31–33]. Assessed by optical coherence tomography (OCT), a non-invasive and readily available tool, most studies have reported a significant reduction in mean RNFL thickness in Mild Cognitive Impairment and AD compared to healthy controls [34]. Results from animal studies have been inconclusive with Perez [35] reporting a non-significant neuronal cell loss in the retina based on retinal layer thickness of APP/PS1 mice, while Liu [36] reported a significant decrease in the thickness of the retina in Tg2576 mice in comparison with WT controls (*P* = 0.0086). Consistent with the latter, our results showed that there was a significant age-related reduction in the inner retinal thickness of APP/PS1 and WT control mice from 3 to 12 months. Furthermore, there was a significant difference between the inner and outer retinal thickness of APP/PS1 and WT mice starting from 9 and 12 months, respectively.

Retinal Aβ deposition in 12 months old APP/PS1 mice has been reported before [37]. More interesting, Koronyo has shown deposition of retinal plaques precede the brain in the APP/PS1 mice (2.5 months vs 5 months) [8]. In this study, we observed retinal Aβ starting from 6 months (vs 3 months in the hippocampus), however, the discrepancy between our findings and the aforementioned report maybe due to the different Aβ staining method used. Koronyo has developed and used a curcumin based compound to visualise Aβ (and its isoforms), whereas we have used the more common Thioflavin S approach which can only visualise neuritic plaques. Thioflavin S, a homogenous dye, selectively binds to the beta-sheets of proteins, and as a result, it undergoes a shift in its emission wavelength upon binding to oligomers. Thioflavin S cannot bind to monomeric isoforms and therefore cannot be detected using fluorescence imaging [38, 39]. We initially observed Thioflavin S positive Aβ in the outer plexiform and inner nuclear layer of the retina. By 12 months of age, Aβ appeared in all of the layers of the inner retina with the majority observed in the GCL.

Previous reports have shown cognitive impairment as a late occurrence of the APP/PS1 mice in the Morris water maze starting from 7 to 8 months [40, 41]. We assessed cognitive impairment using the NOR test. While Zhang [42] has shown that both the NOR and the Morris water maze work equally well in the cognitive evaluation of APP/PS1 mice, however, results from a current meta-analysis [43] suggest that there is no significant association between experimental evaluation of cognitive deficits and quantified cerebral Aβ levels in these animals. Our results also confirm this; we found a non-significant decrease in the recognition index of the APP/PS1 mice from 6 to 12 months of age. Simultaneously, there was a non-significant increase in the recognition index of the WT control mice for the same period.

Our study has a few limitations. First, the animal model used in this study, the double transgenic APP/PS1 mouse model of AD, is a representative of the familial subform of AD that some would refer as a strain only be generalised to the familial subform only. However, our study was aimed at investigating the association between physiological retinal structural and functional parameters with progressive AD-specific cerebral pathophysiological changes. Therefore, our results may be generalised to AD broadly. Furthermore, the number of animal models that replicate the sporadic AD is limited, with the *Octodon degus* as the only animal model of natural AD-specific neuropathology reported in the literature [44]. Second, as the main aim of our study was to identify early retinal changes in AD, therefore we only aged the animals up until 12 months. Given that neuronal loss, based on the phenotype, occurs starting at around 17 months, it might be interesting to age these animals further to determine whether the changes we observed are accelerated past the 17 month time-point. However, most of the current literature has already reported on retinal dysfunctional and structural damage in the established aged animal models of AD and human participants dismissing any further need in a study past the 17 month time-point. Third, the inter-group analysis revealed an exacerbation of retinal functional decline and structural damage in the APP/PS1 group. Given that there was an age-associated decline in both groups, there is a potential for marginal bias in our selected analysis and modelling.

Finally, the OCT device we have used in this study does not have tracking abilities and therefore, intensity/reflectance variability over time may have altered the OCT scans. To address this and ensure OCT scans were taken exactly at the same location, we adopted a few strategies. 1) we segmented the optic nerve head (manually) on the fundus en face and used the B-scan that transected the ONH exactly through the middle of the ONH as reference, 2) the distance between the B-scans, obtained on either side of the ONH, and the reference B-scan was measured in the Image J to ensure consistency at each time point, and 3) to adjust for artefacts due to reflectance, we normalized the intensity measurements of the inner and outer retina to the RPE intensity in each animal at each time point. However, RPE intensity at each time point was very similar, suggesting that reflectance artifact had minimal (if any) effect on our measurements (*p* = 0.2). While the manual segmentation of the ONH may itself lead to subjective errors, however, the combination of strategies employed (i.e. averaging, consistency in measurement locations, and intensity normalizing) minimizes the risk of artefact altering the results.

## Conclusions

In conclusion, we show for the first time that retinal dysfunction precedes retinal structural damage in APP/PS1 mouse model of AD. A significant decline in the b-wave of ERG commenced from 3 months, coinciding with hippocampus Aβ deposition. This suggests that retinal dysfunction occurs in parallel to pathological changes in the brain in AD. Further, we observed a dysfunction of the inner retina starting from 6 months with structural damage to the same region following on from 9 months. We did not observe any cognitive deficit in our cohort, however, this maybe due to the fact that this particular animal model does not show signs of cognitive dysfunction parallel to increased amyloid pathology.

## Additional file

Additional file 1: **Figure S1.** No Aβ was observed in the hippocampus and retina of the WT group for the same time-points. **Figure S2.** Slope analysis (ANCOVA) for Novel Object Recognition test from 6 to 12 months. We observed no significant difference between the two animal groups and across age. (DOCX 1024 kb)

## Data Availability

The dataset supporting the conclusions of this article is available upon a formal and reasonable request from the corresponding author and after the necessary clearances has been obtained from University of Technology Sydney’s technology transfer office.

## Abbreviations

AD: Alzheimer’s disease
APP/PS1: Amyloid Precursor Protein- Presenilin 1
Aβ: Amyloid Beta
ERG: Electroretinogram
GCL: Ganglion Cell Layer
INL: Inner Nuclear Layer
MRI: Magnetic Resonance Imaging
NOR: Novel Object Recognition
OCT: Optical Coherence Tomography
OP: Oscillatory Potential
OPL: Outer Plexiform Layer
PET: Positron Emission Tomography
RGC: Retinal Ganglion Cells
RNFL: Retinal Nerve Fibre Layer
RPE: Retinal Pigment Epithelium
STR: Scotopic Threshold Response
WT: Wild Type
