# Supplementary material for: A longitudinal assessment of retinal function and structure in the APP/PS1 transgenic mouse model of Alzheimer’s disease
Source: Transl Neurodegener. 2019 Oct 1;8:30. doi: 10.1186/s40035-019-0170-z (PMC6774218; doi:10.1186/s40035-019-0170-z)
Supplement: Supplementary file 1 — Figure S1. No Aβ was observed in the hippocampus and retina of the WT group for the same time-points. Figure S2. Slope analysis (ANCOVA) for Novel Object Recognition test from 6 to 12 months. We observed no significant difference between the two animal groups and across age. (DOCX 1024 kb) [file 40035_2019_170_MOESM1_ESM.docx]

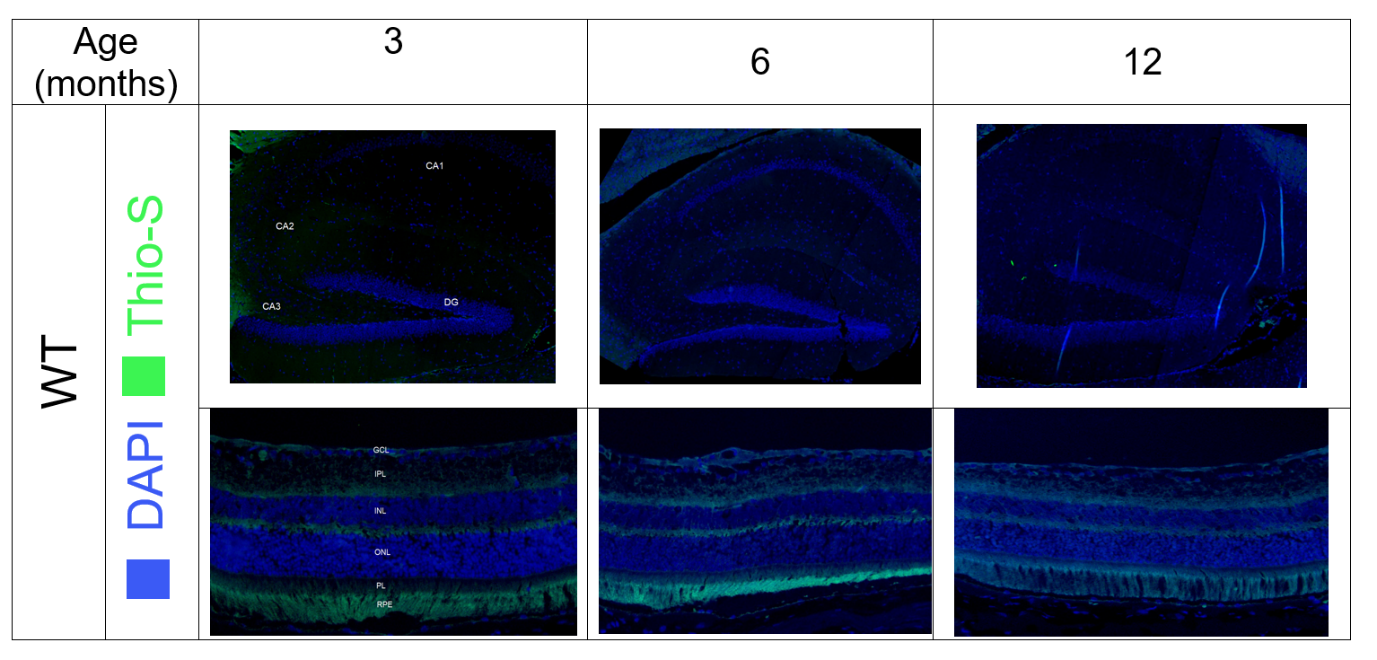


**Supplement Figure 1-** No Aβ was observed in the hippocampus and retina of the WT group for the same time-points.


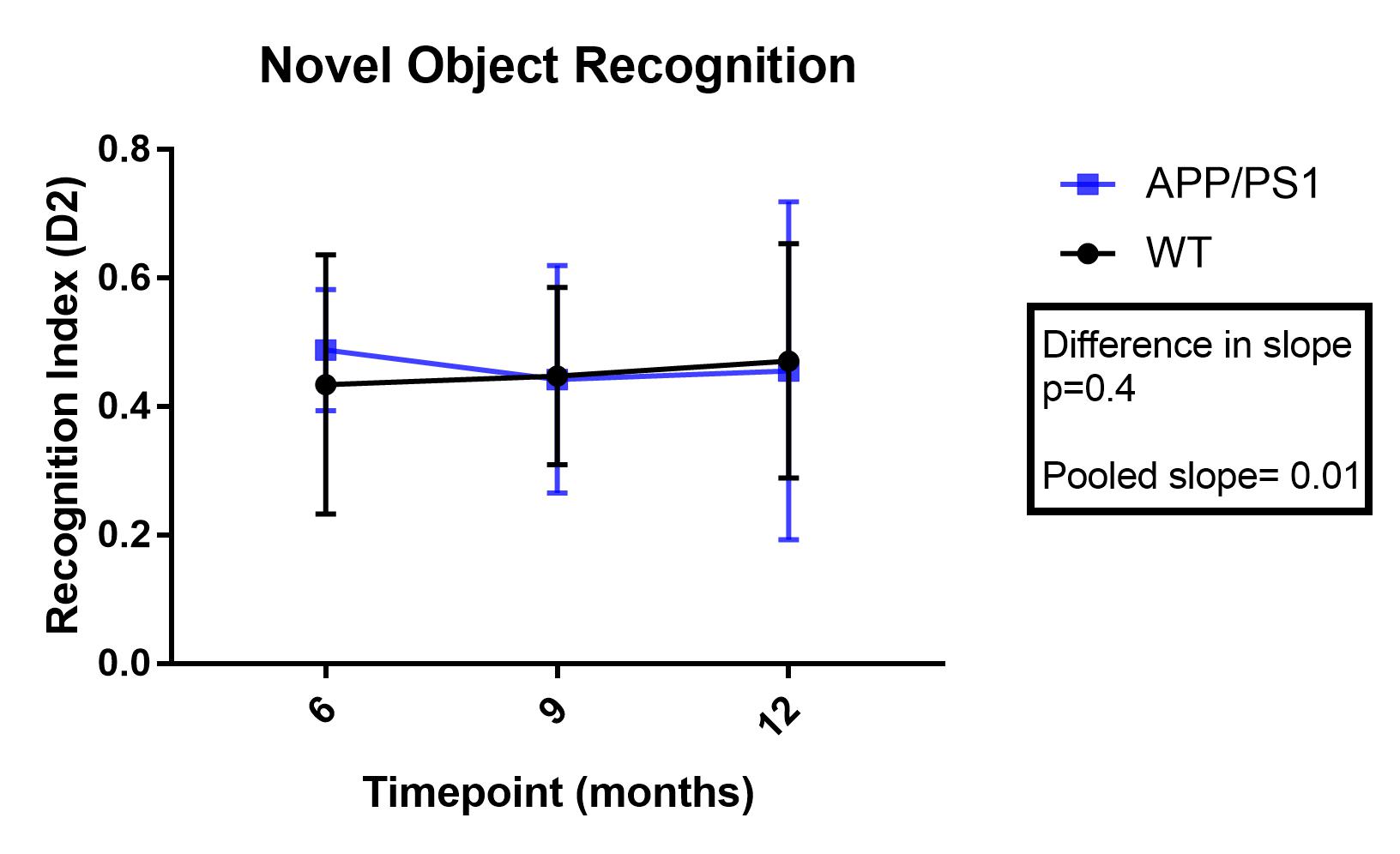


**Supplement Figure 2-** Slope analysis (ANCOVA) for Novel Object Recognition test from 6 to 12 months. We observed no significant difference between the two animal groups and across age.
